# Regional consensus opinion for the management of Beta thalassemia major in the Arabian Gulf area

**DOI:** 10.1186/1750-1172-8-143

**Published:** 2013-09-17

**Authors:** Mohamad H Qari, Yasser Wali, Muneer H Albagshi, Mohammad Alshahrani, Azzah Alzahrani, Ibrahim A Alhijji, Abdulkareem Almomen, Abdullah Aljefri, Hussain H Al Saeed, Shaker Abdullah, Ahmad Al Rustumani, Khoutir Mahour, Shaker A Mousa

**Affiliations:** 1 College of Medicine, King Abdul Aziz University, Jeddah, Saudi Arabia; 2 College of Medicine, SQU, Muscat, Oman; 3 Maternity and Children Hospital, Al Ahsa, Saudi Arabia; 4 Prince Sultan Military Medical City, Riyadh, Saudi Arabia; 5 Clinical Hematology Unit, Department of Medical Oncology, National Centre for Cancer Care and Research, Hamad Medical Corporation, Doha, Qatar; 6 College of Medicine & KKUH, King Saud University, Riyadh, Saudi Arabia; 7 King Faisal Specialist Hospital and Research Center, Riyadh, Saudi Arabia; 8 Department of Medicine, Qatif Central Hospital, Qatif Eastern Province, Saudi Arabia; 9 Princess Noura Oncology Center, King Abdulaziz Medical City, Jeddah, Saudi Arabia; 10 Dubai Health Care City, Dubai, United Arab Emirates; 11 Hopitaux Du Leman, Annemasse, France; 12 The Pharmaceutical Research Institute at Albany College of Pharmacy and Health Sciences, Rensselaer, New York, USA

**Keywords:** Anemia, Chelation, Arabian Gulf, Iron chelation therapy, Iron overload, Thalassemia management, Transfusion

## Abstract

Thalassemia syndrome has diverse clinical presentations and a global spread that has far exceeded the classical Mediterranean basin where the mutations arose. The mutations that give rise to either alpha or beta thalassemia are numerous, resulting in a wide spectrum of clinical severity ranging from carrier state to life-threatening, inherited hemolytic anemia that requires regular blood transfusion. Beta thalassemia major constitutes a remarkable challenge to health care providers. The complications arising due to the anemia, transfusional iron overload, as well as other therapy-related complications add to the complexity of this condition. To produce this consensus opinion manuscript, a PubMed search was performed to gather evidence-based original articles, review articles, as well as published work reflecting the experience of physicians and scientists in the Arabian Gulf region in an effort to standardize the management protocol.

## Introduction

Thalassemia is a group of inherited disorders that arise as a result of certain mutations in hemoglobin (Hb) genes, affecting the makeup of Hb in the red blood cells, which leads to certain pathophysiological disorders [1]. The genetic disorder is characterized by the absolute or partial synthesis of one or more alpha (α)- or beta (β)-globin chains [2,3]. The types of β-thalassemia are called major, intermedia, and minor. β-Thalassemia major is caused by a defect in 2 genes that leads to absence or a severe decrease in β-globin synthesis. β-Thalassemia intermedia is a clinical phenotype with moderate anemia and transfusion independence. Genetically it results from mutations in the 2 β genes resulting in mild to moderate decrease in their synthesis. β-Thalassemia minor, or thalassemia trait, occurs when the defect is present in only 1 gene [2].

β-Thalassemia carriers comprise 1.5% of the worldwide population, with an estimated 60,000 infants with a serious defect being born every year [1]. In the United States, approximately 1,000 individuals have β-thalassemia major, the most severe form of thalassemia [4]. It is most commonly found in people of Mediterranean descent, such as Italians and Greeks, but also affects people from other parts of the world such as Africa, the Middle East, the Indian subcontinent, and Southeast Asia [2,5].

Blood transfusions are often required to treat patients with thalassemia major, who are transfusion-dependent and frequently need to receive 2 to 4 units per month of packed red blood cells [6]. Patients with β-thalassemia major require transfusions throughout life to achieve the target Hb level in the range 9 to 10 g/dL and to promote normal growth [7,8]. Iron overload can develop in transfusion-dependent patients as a result of non-removal of excess iron. High iron concentration load can cause complications due to iron deposition in organs such as the heart, liver, and endocrine glands.

### Epidemiology

Thalassemia is the most common form of inherited anemia worldwide [9]. It occurs with a high frequency in a broad belt extending from the Mediterranean basin through to the Middle East, Indian subcontinent, and Southeast Asia [10]. Thalassemia is a growing global health problem due to extensive population migrations. About 3% of the world population (about 200 million people) are carriers of the β-thalassemia gene [11].

The incidence of β-thalassemia in Arabian Gulf countries is not clearly known due to lack of mandatory screening programs [12-16]. The majority of data were obtained from scattered screening studies using Hb electrophoresis as summarized in Table 1.

**Table 1:** **Estimated prevalence of β**-**thalassemia** (**minor and major**) **in Arabian Gulf countries**

**Country**	**Minor (%)**	**Major (%)**	**Reference**
Saudi Arabia	3.4	NA*	[12]
Bahrain	0.88	0.16	[13]
Qatar	28	0.07	[14]
United Arab Emirates	1.7	NA*	[15]
Oman	2	0.04	[16,17]

* Not available.

### Pathophysiology

β-Thalassemia is a group of heterogeneous autosomal recessive disorders arising due to the absence or reduced synthesis of the β-globin chain [18]. β-Thalassemia could be associated with severe congenital disorders caused by mutations in the *β*-*globin* gene resulting in the absence or reduced synthesis of the β-globin chain [1,12]. This deficit of β-globin chains leads to precipitation of excess α-globin chains resulting in the formation of inclusion bodies, which contribute to hemolysis of red blood cells. Hematological changes are manifested between the ages of 3 months and 6 months onwards [13]. Over 200 different mutations have been elucidated worldwide, and the mutations are population-specific. In the Middle East, codon 39 (C > T), IVSI-110 (G > A), IVSI-1 (G > A), IVSI-6 (T > C), IVSII-1 (G > A), codon 5(−CT), and IVSI-5 (G > C) mutations account for more than 90% of β-thalassemia mutations in the region. However, these mutations differ in numbers and frequencies between different populations of the Middle East. Over 50 different mutations have been identified in the Arab populations, reflecting the heterogeneity of these populations [19,20]. The molecular spectrum of β-thalassemia in the Arab populations of Jordan, Egypt, Syrian Arab Republic, Lebanon, Yemen, and Saudi Arabia revealed that the most frequent mutations were: IVS-1-5 (G–C), IVS-II-1 (G–A), IVS-1-1, Fr 8/9, Fr 41/42, Cd 15, Cd 16, Cap +1 (A–C), IVS-1-110, IVS-1-3′ end (−25 bp) and IVS-1-6 [21-23].

### Presentation and diagnosis

β-Thalassemia comprises a complex spectrum of clinical presentations. The clinical phenotype is influenced by the genetic phenotype and other modifiers. The basic disease is caused by the inability to produce normal β-globin chain with the consequence of excess α-chain, leading to disruption of the α:β ratio, which in turn will cause various cellular and clinical syndromes [1,24].

β-Thalassemia is clinically classified into 4 subtypes (Table 2):

1. β-thalassemia silent carriers; asymptomatic, one β-globin chain is normal and the other is partially defective (ββ^+^).

2. β-thalassemia trait; one β-chain is totally defective and the other is normal (ββ°). These patients have mild anemia, and <20% have palpable spleen [25,26].

3. β-thalassemia intermedia; this term is useful clinically, but does not correlate with its genetic or clinical mechanism for the phenotype [27,28]; usually used to describe β-thalassemia patients who do not require chronic red cell transfusion in early childhood, although by the second decade of life they may present the same picture as β-thalassemia major [28]. Most of the patients have homozygous partial production defect of β globin β^+^β^+^ or β^+^β°, and there are many co-inheritance factors that influence the clinical spectrum (Table 1). Patients with β-thalassemia intermedia frequently develop moderate to severe skeletal changes because of bone marrow expansion, osteoporosis, leg ulcers, gallstones, and pulmonary hypertension and have an increased predisposition to thrombosis as compared to thalassemia major [29-33]. Cardiac involvement in thalassemia intermedia results mainly from a high-output state and pulmonary hypertension, while systolic left ventricle function is usually preserved [31]. These patients have a spectrum of severity, but all have complications in common:

a. Chronic anemia: high cardiac output, increased pulmonary vascular resistance, pulmonary hypertension, and heart failure

b. Iron overload: because the patients are not receiving chronic transfusion, there is increased gut iron absorption with subsequent symptoms and signs of iron overload

Treatment of individuals with thalassemia intermedia is symptomatic [33]. As hypersplenism may cause worsening anemia, retarded growth, and mechanical disturbance from the large spleen, splenectomy is recommended. Prevention of post-splenectomy sepsis includes standard immunization and antibiotic prophylaxis. The gallbladder should be inspected during splenectomy and removed in all cases to prevent gallstones. Treatment of extra-medullary erythropoietic masses, detected by magnetic resonance imaging, is based on radiotherapy, transfusions, or hydroxycarbamide. Leg ulcer requires regular blood transfusions, zinc supplementation, hydroxycarbamide, pentoxifylline, and possibly erythropoietin and platelet-derived growth factor. The high risk of thrombosis, exacerbated by splenectomy, requires proper anticoagulation and/or antiplatelet agents prior to surgical or other high-risk procedures. Chelation therapy is essential to prevent iron overload. In some Arabian Gulf countries, patients with known thalassemia intermedia mutations (e.g., Hb Dhofar) are regularly hyper-transfused from the beginning to avoid the serious complications of excessive medullary and extra-medullary haematopoiesis [34,35].

4. β-thalassemia major (transfusion-dependent β-thalassemia); total impairment of β-globin chain production leading to excess α-globin chain, which in turn is unable to form soluble tetramers and is precipitated in the cell, leading to a sequence of cellular and clinical events. These patients are well at birth, and the clinical picture will start after the fetal Hb (Hb F) switch to adult Hb (Hb A) fails because there is no β-chain produced, leading to anemia, which usually starts after 6 months of age. The severity of phenotype is heterogeneous depending on the type of mutation influencing the β-chain production, e.g., β°, β^+^, the balance between α- and β-chain and Hb F production [27,29,30,36-39]. The manifestations in untreated patients include pallor, irritability, cardiac failure, growth failure, hepato-splenomegaly, bone abnormality, and features of hemolysis. The majority of untreated patients under the age of 5 years die due to anemia, heart failure, or infections [27,40-45].

**Table 2:** **The phenotype and genotypes of β**-**thalassemia**

**Phenotypes**	**Genotype**	**MCV**	**Anemia**	**Hb electrophoresis**
Silent carrier	ββ^+^	Low/Normal	None	Normal
Minor (Trait)	ββ°	Low	Mild	High Hb A_2_
Intermedia	β^+^β^+^ and others	Low	Moderate	Presence of small amount of A and could be similar to Major
Major	β°β°	Low	Severe	Hb A absent, only Hb A_2_ and Hb F are present

*HB* hemoglobin, *Hb A* adult hemoglobin, *Hb F* fetal hemoglobin, *MCV* mean corpuscular volume.

The patients who have severe β-thalassemia develop symptoms in the first year of life with progressive anemia, failure to thrive, poor feeding, intermittent bouts of infection, and general malaise in most cases. These infants are pale and, in many cases, splenomegaly is already present. A few patients might present as late as between the third to fifth year of age [27,30,46]. The diagnosis of β-thalassemia requires a patient history, physical examination, and confirmatory laboratory tests including complete blood count and blood film morphology, both demonstrating microcytic hypochromic anemia, and confirmation by alkaline Hb electrophoresis (Table 2) [46].

The features of the different presentations of thalassemia syndromes are:

1. β-thalassemia silent carrier

•Normal phenotype

•Laboratory: normal Hb, RBC indices, and Hb electrophoresis

2. β-thalassemia minor (trait)

•Haematocrit >30%

•Hb and peripheral morphology similar to iron deficiency state

•RBC count more than normal, MCV is low

•RDW is normal

•Hb electrophoresis or HPLC show elevated Hb A_2_ (except in rare cases will be normal)

3. β-thalassemia intermedia

•Hb is low (6-10 g/dL)

•CBC and peripheral morphology are variable with hypochromic, microcytic changes with variation in RBC size and shape

•RDW is increased

•Hb electrophoresis or HPLC show different patterns; some patients are able to produce Hb A in small quantities or similar to homozygous β-thalassemia zero where Hb A is absent

4. β-thalassemia major

•CBC:

◦False elevated WBC because of nucleated RBC

◦Severe anemia with Hb as low as 3-4 g/dL

◦Microcytic hypochromic picture with fragmented RBC, tear drops, target cells, and RBC with inclusion bodies

•Reticulocyte count low

•Serum iron to TIBC is high

•Hb electrophoresis: absent Hb A with remaining as Hb F and Hb A_2_ (Table 2)

The final step in a diagnostic approach is to perform DNA studies on the genetic material extracted from peripheral blood. This will identify the type of the mutation and help identify the silent carriers [18].

### Transfusion therapy in thalassemia

Red cell transfusions are required to increase the oxygen-carrying capacity of the blood through raising the Hb concentration of patients with acute or chronic anemia [1]. Guidelines for the transfusion of blood and blood components and the management of transfused patients are in accordance with the British Committee for Standards in Haematology [47]. The major goals for blood transfusion therapy include [48,49]:

1. Maintenance of red cell viability and function during storage to ensure sufficient transport of oxygen

2. Use of donor erythrocytes with a normal recovery and half-life in the recipient

3. Achievement of appropriate Hb level

4. Avoidance of adverse reactions, including transmission of infectious agents.

### Current practice and recommendation for transfusion therapy in Arabian Gulf countries

The recommended treatment for thalassemia major involves lifelong, regular blood transfusions, usually administered every 2 to 5 weeks to maintain the pre-transfusion Hb level above 9.5-10.5 g/dL [50,51]. However, in most of the Gulf countries, 9 g/dL is accepted as the minimum pre-transfusion Hb level. Higher levels (11–12 g/dL) may be needed for patients with cardiac complications. The post-transfusion Hb is kept not higher than 14–15 g/dL.

Our practice includes extended red cell antigen typing of patients including C, E, and Kell before the first transfusion. At each transfusion, we do a full cross match and screening for the new antibodies. Matching for C, E, and Kell antigens is only done for negative patients. The blood is leuko-reduced with pre-storage filtrations. Some countries also practice bed-side filtrations. Transfusion is attended by nurses who report any adverse reactions. Blood that has not been transfused by 4 hours after hooking is discarded.

### Iron overload

During the introduction of a regular transfusion program in the early 1960s, an iron chelating agent demonstrated improved survival. However, iron overload is still one of the most critical issues, and its complications remain the most important cause of morbidity and mortality [52,53]. In addition, the lack of adherence to an iron chelating agent regimen is considered an important factor in suboptimal clinical improvement and poor prognosis [54]. Many significant developments have been made in the assessment of iron overload, and advances in the modalities available for iron chelation have expanded treatment options and improved treatment management.

In a chronically transfused patient, one unit of blood contains 200 mg iron [55]. Since humans have no physiologic mechanism for active elimination of excess iron, patients receiving regular RBC transfusions develop cumulative iron overload and are at risk for iron toxicity [55].

In addition to the transfused iron, thalassemia patients absorb more iron than normal individuals. The mechanism of increased absorption in thalassemia patients is thought to be related to paradoxical Hepcidin suppression from dys-erythropoiesis [56]. Free iron is subsequently deposited primarily in parenchymal cells of different organs. Then it will participate in oxidative reactions to generate free oxygen radicals, which can lead to chronic cell toxicity and DNA damage [57].

### Measurements of iron load

Parameters used to monitor iron load include serum ferritin, liver biopsy, and MRI assessment of liver and cardiac iron, in conjunction with functional testing such as echocardiography, liver function test, and measures of endocrine function.

#### Serum ferritin

Serum ferritin is the most commonly used parameter for monitoring iron overload despite its inaccuracy and limitation in assessing the body iron overload. It is a valuable method to roughly assess the long-term, overall status of iron overload and to monitor response to chelation therapy. It is also valuable due to its ease of measurement and wide availability, and it correlates with cardiac impairment and survival but not with hepatic iron [58]. Serum ferritin is elevated in many other conditions such as infections, inflammation, or malignancy. Iron overload is generally defined as serum ferritin consistently ≥ 1000 ng/L [59]. It is recommended to know the baseline level of ferritin and to assess the trend by taking serial measurement of serum ferritin every 3 months. In the Gulf area, due to the wide availability of the test in all treating centers, the test is done on a monthly basis.

#### Liver biopsy

Liver iron concentration remains the most accurate measure of total body iron loading [60], and liver biopsy was previously considered to be the gold standard of liver iron assessment, but is an invasive procedure associated with a risk of complications. Liver biopsy is still performed to evaluate liver fibrosis, cirrhosis, or hepatocellular carcinoma, which are possible complications in all patients with liver iron overload. It is well known that a hepatic iron level of 7–15 mg/g dry weight is associated with an increased risk of complications; a higher level increases risk of cardiac disease and early death. However, because of its invasive nature and lack of cultural acceptance, this test is rarely performed in the Gulf area centers.

#### MRI assessment of liver iron

MRI is the method of choice in the monitoring of various organs’ iron levels where the measurement of tissue proton transverse relaxation rates (R2) was shown to have excellent correlation with liver iron concentration measured by biopsy [61]. An annual monitoring of R2 MRI is recommended, which can be extended to every 2 years for patients with normal liver iron or at the lower end of the ideal iron range of less than 7 mg/g dry weight. Additionally, liver iron levels should also be correlated with standard liver function tests.

#### MRI assessment of cardiac iron

Similar to liver iron measurement, MRI has been widely used to annually monitor iron levels in the heart where the measurements of T2*, a relaxation parameter intrinsic to protons placed in the magnetic field, is utilized [62]. In the Gulf region, very few centers have this tool available. It is recommended to expand this service for thalassemia patients because MRI is a noninvasive, reliable, and accurate method of assessing iron overload.

### Chelating agents

There are 3 main available chelators: deferoxamine, deferiprone, and deferasirox (Table 3); deferiprone and deferasirox are oral chelators that have come into the clinic in recent years. They are different in molecular weight, leading to differences in intestinal absorption. For several decades, the only available iron chelator was deferoxamine [58].

**Table 3:** Comparative analysis of different iron chelators

	**Deferoxamine (DFO)**	**Deferiprone (DFP)**	**Deferasirox (DFX)**
**Brand name**	Desferal	Ferriprox	Exjade
**Chelator-iron, complex ratio**	Hexadentate, 1:1	Bidentate, 3:1	Tridentate, 2:1
**Dose (mg/kg/day**)	25–50	75–100	20–40
**Combination and titration doses (mg/kg/day)**	Combination therapy with DFO and DFP, 2 days/week DFO and then continue with DFP		Titration therapy
**Administration**	Subcutaneous or intravenous, 8–10 hrs/day, 5–7 days/wk	Oral, 3 times daily	Oral, once daily
**Plasma half-life (hr)**	0.5	2–3	8–16
**Route of elimination**	Biliary and urinary	Urinary	Biliary
**Regulatory approval**	US, Canada, Europe, other countries	US, Europe, other countries	US, Canada, Europe, other countries
**Indication**	Transfusion iron overload and acute iron overload	Transfusion iron overload when DFO is contraindicated or inadequate	Transfusion iron overload
**Adverse events**	Irritation at the infusion site, ocular and auditory disturbances, growth retardation and skeletal changes, allergy, respiratory distress syndrome with higher doses	Agranulocytosis and neutropenia, gastrointestinal disturbances, arthropathy, increased liver enzyme levels, low plasma zinc level, hepatic fibrosis	Gastrointestinal disturbances, rash, increase in serum creatinine level; potentially fatal renal impairment or failure
**Advantage/ Disadvantage**	Inexpensive/ Compliance	Route of administration / Compliance	Route of administration / Expensive

In the Gulf region, deferoxamine has been available since the early 1970s, and nowadays it is much less widely used except in combined chelation therapy. In 1999, deferiprone was introduced to Oman and has been widely used as monotherapy or combined therapy. In other Gulf States it was introduced in early 2000s. Although the international guidelines recommend the use of deferiprone after the age of 6 years, in some Arabian Gulf countries the syrup formulation has been widely used with good efficacy for children 2 years and above with no serious complications. In a recent study, continued deferiprone therapy during episodes of mild neutropenia (down to 1×10^9^/L) has not been associated with progression to agranulocytosis [63]. We recommend giving extensive counseling to the patients, with clear instructions to report to hospital emergency in case of fever and to have an urgent CBC with ANC count.

Deferasirox has been available in the Gulf area since 2007. It has been widely used across all the Gulf countries. Side effects are comparable (Table 3) to those published in the literature [64], and its efficacy has been demonstrated in many clinical trials [65]. It is used only as monotherapy. Some patients do not respond to the maximum dose of 40 mg/kg/day, however, and compliance may be an issue with this drug, just as it is for the other available chelators.

### Hematopoietic stem cell transplantation (HSCT) for thalassemia

HSCT has the potential to be curative for thalassemia major and has been increasingly adopted, with cautions. The first two cases of HSCT for thalassemia were performed in 1981, one in Seattle, Washington, and the other in Pesaro, Italy [66]. Since 1981, a large clinical experience has been gained with more than 2,000 HCSTs in centers around the world. The patient classes have been identified on the basis of 3 risk factors (the Pesaro classification): inadequate iron chelation therapy, presence of liver fibrosis, and hepatomegaly (Table 4) [67].

**Table 4:** Stem cell transplantation for thalassemia

	**Class I**	**Class II**	**Class III**
**Number of risk factors**	None	1-2	3
**Survival (%)**	93	87	79
**Event free survival (%)**	91	83	58
**Rejection (%)**	2	3	28
**Risk of transplant**-**related mortality (%)**	8	15	19
**Risk of transplant**-**related morbidity (%)**	9, mainly GVHD	17	22

*GVHD* graft-versus-host disease.

#### Factors to be taken into consideration for HSCT in thalassemia

#### Age

Since gonadal damage occurs with the myeloablative conditioning regimens currently used in thalassemia transplants, it is important to try to perform transplant as early as possible to minimize infertility. An age range of 2–5 years may seem reasonable [68]. For several reasons an earlier patient age at transplant has a better outcome because a fewer number of transfusions (allosensitization) would decrease the chances of rejection, and complications of iron overload and organ and transplant-related toxicity will be less. Accordingly, tolerance for myeloablative conditioning regimens would be better compared with older age groups and adults [68,69].

#### Conditioning

The standard conditioning regimen that is practiced in most HSCT units all over the world, especially for class I and II patients, consists of busulfan and cyclophosphamide and GVHD prophylaxis in the form of cyclosporine A and methotrexate [70,71]. New developments in conditioning regimens have taken place over the last decade. The introduction of IV busulfan with drug level monitoring and IV fludarabine improved the efficacy and reduced the toxicity of transplant outcome [72]. In addition, HSCT outcome for patients of older age with advanced disease receiving a second transplant improved markedly with improved pre-transplantation preparation and simultaneous use of myeloablative and prolonged immune suppression. In order to be utilized effectively and to minimize toxicity and rejection, it is important that busulfan levels are monitored and doses adjusted accordingly [72,73]. Several centers have recently reported using new therapeutic conditioning regimens utilizing the combination of treosulfan, thiotepa, and fludarabine because treosulfan does not require therapeutic drug monitoring. The outcome of the new regimen may be effective and safer even for more advanced thalassemia and may replace busulfan as the conditioning of choice [74,75].

#### Matched umbilical cord as stem cell source

While HSCT from a bone marrow harvest of a fully HLA-matched donor is the recommended option, genetically matched umbilical cord blood can be a suitable source [76]. This provides an alternate source of stem cells with minimal risk to the donor. In the Gulf area, experience with this form of HSCT is still very limited.

#### Results of HSCT for thalassemia in the Gulf area

Two countries are doing HSCT, namely Oman and Saudi Arabia. Between 1996 and 2010, a total of 47 patients with thalassemia major underwent matched sibling donor HSCTs in Oman. Most received busulfan and cyclophosphamide with or without ATG, while more recently patients received busulfan and fludarabine. Eighty-nine percent are alive and free from thalassemia at a median follow-up of 125 months (22–197 months). Graft rejection was 8.5%, and transplant-related mortality was 4.3% [77].

In Saudi Arabia, transplants in 60 thalassemic patients were done between January 1998 and July 2006 from HLA-related, matched donors. The overall survival and event-free survival were 94% and 77%, respectively [69].

### Complications in thalassemia

Iron overload can be either from thalassemia itself or from frequent blood transfusions. The damage is characterized by excessive iron deposition without adequate iron chelation therapy; almost all patients with β-thalassemia will accumulate potentially fatal iron levels [78]. A range of complications including endocrinopathies, hypersplenism, infertility, hepatobiliary, musculoskeletal, and cardiopulmonary can arise. Complications are linked to overstimulation of the bone marrow, dysfunctional erythropoiesis, an increase in iron burden, imbalance of oxidant/antioxidant ratio, and chelation of essential elements such as zinc.

#### Endocrinopathies

Endocrinopathies are characterized by poor growth; delayed puberty, infertility, and impaired glucose tolerance are some complications that can occur in thalassemia patients [79-81]. Hypogonadism is a common complication that occurs due to a high iron burden [82-85]. Endocrine complications arise possibly due to factors that influence the anterior pituitary gland, which might be due to free radical oxidative stress damage for the pituitary and hypothalamus leading to growth hormone deficiency, resulting in delayed growth and infertility [85,86].

Early and appropriate management is crucial in β-thalassemia patients, and hormone therapy is an effective option in the management of hypogonadism [87]. Hormone therapy to young β-thalassemia patients with low iron burden can induce increases in height, growth, and stimulate puberty [83,88]. Initiating aggressive and appropriate chelation therapy is important along with hormone therapy to minimize iron deposition in the endocrine glands. Along with hormone therapy, zinc supplementation is recommended in patients who have growth impairment and low serum zinc levels [85,89].

#### Hypersplenism

Hypersplenism is another complication in patients who have either thalassemia major or thalassemia intermedia, leading to compromised immune function. It is associated with leukopenia, thrombocytopenia, and increased requirement for transfusions. Splenomegaly (enlarged spleen) develops in these patients, often requiring a splenectomy, which is conducted to reduce the need for red cell transfusion [89-92].

#### Infertility/pregnancy

In women with β-thalassemia, infertility is a common issue due to iron deposition and oxidative damage to endocrine organs as a result of blood transfusions. However, with advances in the management of thalassemia, chances of conceiving have increased and pregnancy has become more prevalent, but pregnancy is not recommended in women who have cardiac dysfunction [93]. For women who are at high risk of iron overload and who intend to conceive, an echocardiography is recommended before conception and during pregnancy to evaluate cardiac function [94].

Management of pregnant women with thalassemia major requires blood transfusions and maintaining a Hb level of at least 10 g/dL [93]. According to the American Congress of Obstetricians and Gynecologists (ACOG) guidelines, iron chelation therapy with deferoxamine is not recommended during pregnancy because its safety has not been well established [93,95]. For patients who are at high risk of increased iron deposition in the heart and liver, oral chelation therapy should be used with caution [95]. Administration of deferasirox to animals during pregnancy and lactation resulted in reduced offspring viability [96]. It is important to note that the oral chelator deferiprone is a fairly small molecule that can cross the placenta, and therefore it is contraindicated in pregnancy [97]. After pregnancy, patients can resume iron chelation therapy.

#### Hepatobiliary disorders

It has been documented that within 2 years of transfusion initiation, hepatocellular injury can occur when the liver iron concentrations exceed normal levels, leading to tissue damage, collagen formation, and portal fibrosis [98]. Furthermore, in the presence of infections such as hepatitis C virus infection, there is increased risk of the development of liver fibrogenesis. Reducing iron burden would reduce liver fibrosis and improve survival in β-thalassemia patients since liver iron concentrations greater than 7 mg/g dried weight are shown to be linked to increased morbidity and mortality [98]. Furthermore, β-thalassemia patients might potentially benefit from combination therapy with iron chelators and green tea extract to decrease the progression of liver fibrosis [99].

#### Musculoskeletal

Patients with thalassemia may exhibit dramatic skeletal abnormalities, frequently leading to marked structural changes and delayed skeletal maturation, due to the expansion of erythroid bone marrow, which widens the marrow spaces, attenuates the cortex and produces osteoporosis. The skull and facial bones are often strikingly abnormal, there is prominent frontal bossing, delayed pneumatization of the sinuses, and marked overgrowth of the maxillae. Premature fusion of the epiphyses can result in characteristic shortening of the limbs, particularly the arms. Of equal concern is the thinning of the cortices due to marrow expansion, which often results in pathologic fractures [100].

Bone disease in thalassemia is also attributable to additional factors including iron deposit in bone, vitamin D deficiency, and deferoxamine toxicity in bone. The interactions among iron, hemopoietic cells, osteoblasts, and osteoclasts in bone tissue have never been explained [101,102]. There is now a growing awareness that many transfusion-dependent adult patients with β-thalassemia major and intermedia suffer from long-standing bone pain, low bone mass, and fractures.

Current transfusion and chelation practices might be insufficient to prevent the development of low bone mass, and additional strategies to improve bone mineral density are required. There is a need for clinical trials to determine the appropriate form of hormone replacement therapy along with vitamin D supplementation as other strategies to optimize bone accrual in this disease. In thalassemia patients with iron overload, regular assessment of bone density from puberty every 2 years if normal, and annually if abnormal, might be required. Additionally, it is necessary to ensure adequate calcium intake, sun exposure, and vitamin D intake. Bisphosphonate therapy may be indicated if osteoporosis is documented [103].

#### Cardiopulmonary

Cardiopulmonary dysfunction represents one of the most under-diagnosed complications. The main cardiac abnormalities reported in patients with thalassemia major and iron overload are left ventricular systolic and diastolic dysfunction, pulmonary hypertension, arrhythmias, and pericarditis. These cardiac abnormalities are closely related to concomitant endocrine deficiencies, thromboembolic events, and inflammatory milieu [104,105].

Iron-induced cardiomyopathy is slowly progressive, and it usually takes several decades for clinical or laboratory features of cardiac dysfunction to manifest [106-108]. Cardiac complications represent the leading cause of mortality in thalassemia major with uncontrolled iron overload. Following the introduction of chelating therapies, an important and progressive increase of life expectancy mainly due to improvement of cardiac dysfunction has been demonstrated [106-108].

Pulmonary impairment is shown in a great proportion of patients, even among asymptomatic young patients, thus, regular screening of pulmonary function should be adopted in the routine clinical follow-up of these patients [109]. Pulmonary hypertension is common in thalassemia and contributes to mortality. Advancing age and a history of splenectomy are major risk factors in this population. Guidelines for the management of pulmonary hypertension in thalassemia have not yet been established, however, clinical trials are ongoing in an effort to guide future therapy [90,110,111].

### Psychosocial impact

Psychosocial and behavioral problems are common in thalassemia patients and their family, suggesting the importance of lifelong psychosocial support [112]. Based on the conclusions of previous studies, it was recommended that medical therapy of these patients should be supported with psychological and psychiatric therapy. Chronicity is a powerful source of emotional problems that intensify at each significant developmental stage of the patient’s life. Patients can feel that they are different, limited, or isolated. Their state of mind can shift rapidly from depression to anger and vice versa. Health workers must be prepared to accept this shift and to help patients deal with these feelings and to find their own way of normal life [113,114]. Several objectives have to be achieved to reach the goal of optimal psychosocial integrity of the patients, including:

1. Educate patients and parents to understand the magnitude of the illness.

2. Provide support to patients to be able to take care of themselves.

3. Facilitate a normal lifestyle and encourage patients in ways to develop a normal adult life.

These objectives can be achieved by the support of a well-trained, multidisciplinary team who understands chronic hereditary diseases.

### Future treatment options and prevention

#### Stem cell transplantation

Improvement in the life expectancy of patients with β-thalassemia major is due to effective transfusion and iron chelation therapy. However, the only current curative treatment is stem cell transplantation, which is becoming more utilized in good patient candidates, and several centers are doing it in the Gulf States–in particular Saudi Arabia. Production of fetal Hb by cytotoxic agents such as demethylation agents and hydroxyurea, has been explored over the last two decades [115,116].

#### Prevention

Prevention of thalassemia is the only solution to efficiently reduce the huge medical, social, and economic impact in countries where it occurs with high frequency. Premarital screening became mandatory for hemoglobinopathies in Saudi Arabia with the intention to decrease the prevalence of the disease [115,117]. Pre-implantation genetic diagnosis is an important option for couples at risk of having children with β-globin mutations [118]. Some premarital and antenatal screening centers in the Gulf area refrain from aborting fetuses owing to religious and cultural reasons. Public awareness and related social activities are important tools to improve the understanding of the burden of this disease and of how to support preventive programs aiming to eradicate this disease.

## Conclusions

The prevalence of thalassemia syndrome in the Arabian Gulf area necessitates more work to estimate the size of this health problem and its impact on the community and the quality of life of the patients and their families. The rationale behind the interest in thalassemia is its responsiveness to treatment with regular blood transfusion and the reversibility of complications with iron chelation. In the Gulf States there is a great potential for improving the quality of care provided to thalassemia patients, hence this manuscript was prepared to emphasize the importance of neonatal screening programs.

Efforts of the health care providers should be directed to improve the early diagnosis and management of β-thalassemia through enrolling the patients in a Thalassemia Center of Excellence to ensure the maximum service and to prevent disease complications. Pre-implantation genetic diagnosis is an option that has become widely accepted religiously and socially to prevent severe β-thalassemia in the Gulf Countries.

## Competing interests

The authors declare that they have no competing interests.

## Authors’ contributions

The authors had 3 meetings over a 9 month period to summarize the evidences based on published data and to bring their expertise in the management of thalassemia patients. All authors contributed to the writing and integration of the different segments of the manuscript. All authors read and approved the final manuscript.
